# Lower serum insulin-like growth factor 1 concentrations in patients with chronic insomnia disorder

**DOI:** 10.3389/fpsyt.2023.1102642

**Published:** 2023-04-21

**Authors:** Yanan Zhang, Qingqing Sun, Huimin Li, Dong Wang, Ying Wang, Zan Wang

**Affiliations:** Sleep Center, Department of Neurology, The First Hospital of Jilin University, Chang Chun, China

**Keywords:** chronic insomnia disorder, anxiety, insulin-like growth factor-1, sleep quality, polysomnography

## Abstract

**Objectives:**

Insulin-like growth factor 1 (IGF-1) is a crucial neurotrophin that is produced in the brain and periphery and may play an important role in insomnia and mood disorders. We aimed to analyze its serum concentrations in patients with chronic insomnia disorder (CID).

**Methods:**

Patients with CID were enrolled in this study and divided into the CID group [Generalized Anxiety Disorder-7 (GAD-7) score < 10] and the CID with anxiety group (GAD-7 score ≥ 10). Age-and sex-matched healthy volunteers were recruited as controls. The Pittsburgh Sleep Quality Index (PSQI) was used to assess sleep quality and the GAD-7 and the Patient Health Questionnaire-9 to assess emotional status. All subjects were monitored via polysomnography, and the serum IGF-1 concentrations in their peripheral blood were detected via enzyme-linked immunosorbent assays.

**Results:**

We enrolled 65 patients with CID (of whom 35 had anxiety) and 36 controls. The PSQI score and IGF-1 concentration in the CID and CID with anxiety groups were higher than those in the control group. The apparent difference in IGF-1 concentration between the CID and CID with anxiety groups was not statistically significant. The IGF-1 concentration in patients with CID was linearly correlated with the GAD-7 score, PSQI score, and stage 3 non-rapid eye movement (stage N3) time.

**Conclusion:**

The serum IGF-1 concentration in patients with CID was lower than that of participants without CID, negatively correlated with anxiety score and sleep quality, and positively correlated with stage N3 time.

## 1. Introduction

Sleep is a basic physiological requirement, essential to human life. Sleep disorders are common and have detrimental effects on people’s daily lives, learning, and work. Chronic insomnia is a common sleep disorder, affecting approximately 30% of the general population (1). It is characterized by persistent difficulty in falling asleep or sleep maintenance and resulting in insufficient sleep satisfaction, persisting for at least 3 months (2). Patients with chronic insomnia frequently experience a variety of nervous system symptoms, including fatigue, impaired focus, and excessive daytime sleepiness, which can increase mental disease, immunological dysfunction, and endocrine dysfunction for a long time (2–4).

Sleep is regulated by neuroendocrine signals and associated with the optimal production of hormones, including growth hormone (GH) and insulin-like growth factor 1 (IGF-1) (5, 6). Sleep deprivation and sleep restriction affect endocrine secretion; they increase cortisol concentrations in the evening and decrease concentrations of the anabolic hormone testosterone, GH, and IGF-1 (7, 8). IGF-1, a peptide hormone consisting of 70 amino acids, is a neurotrophic factor that mediates the effects of GH. IGF-1 affects metabolism, cognition, neuroprotection, regeneration, and functional plasticity (9, 10).

The IGF-1 concentration is the recommended biomarker for the diagnosis of growth-related diseases because it does not exhibit short-term variations to the same extent as GH. In mammals, adequate sleep increases the circulation of IGF-1 and insufficient sleep decreases the concentration of IGF-1 in the muscles (11–13). To the best of our knowledge, the IGF-1 concentration in patients with chronic insomnia remains unclear.

The aim of this study was to assess serum concentrations of IGF-1 in patients with chronic insomnia. We hypothesized that patients with insomnia have a decreased serum IGF-1 concentration, which may play a role in the endocrine system, providing more evidence for the evaluation and treatment of insomnia.

## 2. Methods

### 2.1. Participants

We prospectively enrolled patients diagnosed with chronic insomnia disorder (CID) according to the International Classification of Sleep Disorders—Third Edition as outpatients of the Department of Neurology, First Hospital of Jilin University, from March to July 2022. At the time of enrollment, none of the patients were using actual sleeping medication. The inclusion criteria were as follows: (1) age between 18 and 68 years, (2) sleep latency >30 min, (3) difficulty staying asleep, (4) waking up early, (5) daytime sleepiness, (6) impaired daytime function, (7) sleep difficulties and daytime function impairment lasting more than 3 days a week for more than 3 months, and (8) a Patient Health Questionnaire-9 (PHQ-9) score < 10. The exclusion criteria were as follows: (1) intracranial tumors, stroke, intracranial infection, brain trauma, and other central nervous system diseases; (2) diabetes, pituitary diseases, and other systemic diseases; and (3) taking medication that affect the secretion of growth hormones (bromocriptine, progesterone, etc.). These patients were divided into two groups according to their Generalized Anxiety Disorder-7 (GAD-7) score: the CID group (GAD-7 score < 10), and the CID with anxiety group (GAD-7 score ≥ 10). Sex-and age-matched healthy volunteers were recruited as controls. Demographic and clinical variables, including age, sex, marital status, body mass index (BMI), and insomnia duration were recorded.

This study was approved by the Ethics Committee of the First Hospital of Jilin University and followed the guidelines of the Declaration of Helsinki (1964). All the participants or their guardians provided written informed consent for participation in the study.

### 2.2. Questionnaires

Participants self-assessed their sleep quality using the Pittsburgh Sleep Quality Index (PSQI) questionnaire, which is one of the most widely validated and practical sleep disorder assessment scales, mainly used to provide a subjective measure of sleep quality in the last month. The questionnaire consists of 23 items grouped into seven components: subjective sleep quality, sleep latency, sleep duration, habitual sleep efficiency, sleep disturbances, use of sleep medications, and daytime dysfunction (14). Each component is scored from 0 (good sleep quality) to 3 (bad sleep quality), and the total PSQI score (ranging from 0 to 21) is obtained by summing the seven component scores. A total PSQI score ≤ 5 is associated with good sleep quality and > 5 with poor sleep quality. Higher sleep scores on the PSQI scale equate to poorer sleep quality (15).

Severity of anxiety was self-evaluated using the GAD-7 scale (16). The GAD-7 is a seven-item screening instrument, with each response scored from 0 (never) to 3 (almost every day). The total GAD-7 score ranges from 0 to 21, with a higher GAD-7 score indicating higher level of anxiety. A score ≥ 10 on the GAD-7 is the cutoff point for possible anxiety, with a sensitivity of 89% and specificity of 82% (17).

The PHQ-9, an effective self-rating depression scale, was used to assess depression symptoms. Participants rated depression symptoms of the past 2 weeks on a four-point scale, ranging from 0 (never) to 3 (almost every day). The total score ranges from 0 to 27, with higher scores reflecting greater levels of depression symptoms (18). A score of ≥10 on the PHQ-9 is the cutoff point for depression symptoms, with a sensitivity of 80% and a specificity of 92% (19).

### 2.3. Polysomnography

Before polysomnography (PSG), the participants were not permitted to consume caffeinated beverages, alcohol, or sleep medicines. PSG (Compumedics, Abbotsford, Australia) was performed in a standard, sound-attenuated sleep laboratory at our hospital, and participants were monitored for at least 8 h. These studies followed the American Academy of Sleep Medicine (AASM) standards for electroencephalography, electrooculography, chin muscle electromyography, electrocardiography, nasal pressure, finger oximetry, chest, and abdominal respiratory inductance plethysmography. Professional sleep technicians certified as PSG technologists analyzed the PSG results using the AASM version 2.3 for the Scoring of Sleep and Associated Events.

### 2.4. IGF-1 measurements

The IGF-1 concentration was measured in the morning under fasting conditions for all participants. Blood samples were collected at 8 a.m. Blood was centrifuged at 3,000 × *g* for 10 min, and the serum was stored at −20°C before further use. The IGF-1 concentration was measured using the Human IGF-1 Quantikine enzyme-linked immunosorbent assay, which has a measurement range of 2–1,200 ng/mL.

### 2.5. Statistical analysis

Statistical analysis was performed using IBM SPSS Statistics for Windows version 23.0 (IBM Corp., Armonk, NY, United States). The Shapiro–Wilk test was used to assess the normal distribution of continuous variables. Continuous data with a normal distribution [age, BMI, insomnia duration, GAD-7 score, PHQ-9 score, PSQI score, IGF-1 score, total sleep time, sleep latency, sleep efficiency, rapid eye movement (REM) sleep, stage 1 non-REM (stage N1), stage 2 non-REM (stage N2), stage 3 non-REM (stage N3), stage N1 time, stage N2 time, stage N3 time, REM sleep latency, REM arousal index, arousal index, apnea-hypopnea index (AHI), periodic limb movement index (PLMI), mean SaO_2_, minimum SaO_2_, and end-tidal CO_2_] were expressed as means and standard deviations and compared among groups by using one-way ANOVA. Categorical data (male sex and marital status) were expressed as absolute values and percentages and compared among groups by using the chi-square test. The correlations of IGF-1 concentration with the GAD-7 score, PSQI score, and stage N3 time were examined using the Pearson correlation test. Univariate and multivariate linear regression were used to assess the association between IGF-1 and clinical parameters including sex, age, BMI, insomnia duration, GAD-7 score, PHQ-9 score, PSQI score, total sleep time, sleep latency, sleep efficiency, wake after sleep onset, stage N1 time, stage N2 time, stage N3 time, REM sleep time, REM sleep latency, REM arousal index, arousal index, AHI, PLMI, mean SaO_2_, minimum SaO_2_, and end-tidal CO_2_. In the *post hoc* analysis, the Bonferroni method was used to calculate the adjusted *p*-value. The statistical significance level was set to *p* < 0.05.

## 3. Results

### 3.1. Baseline characteristics and IGF-1 serum levels

**Figure 1:**
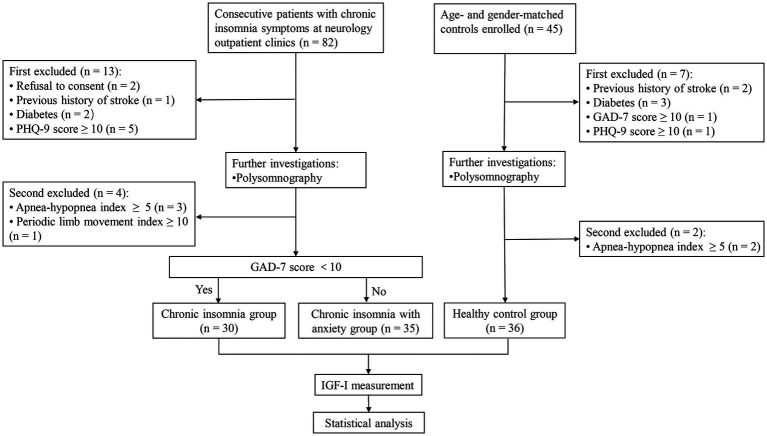
Flowchart shows the screening process for participants.

**Figure 2:**
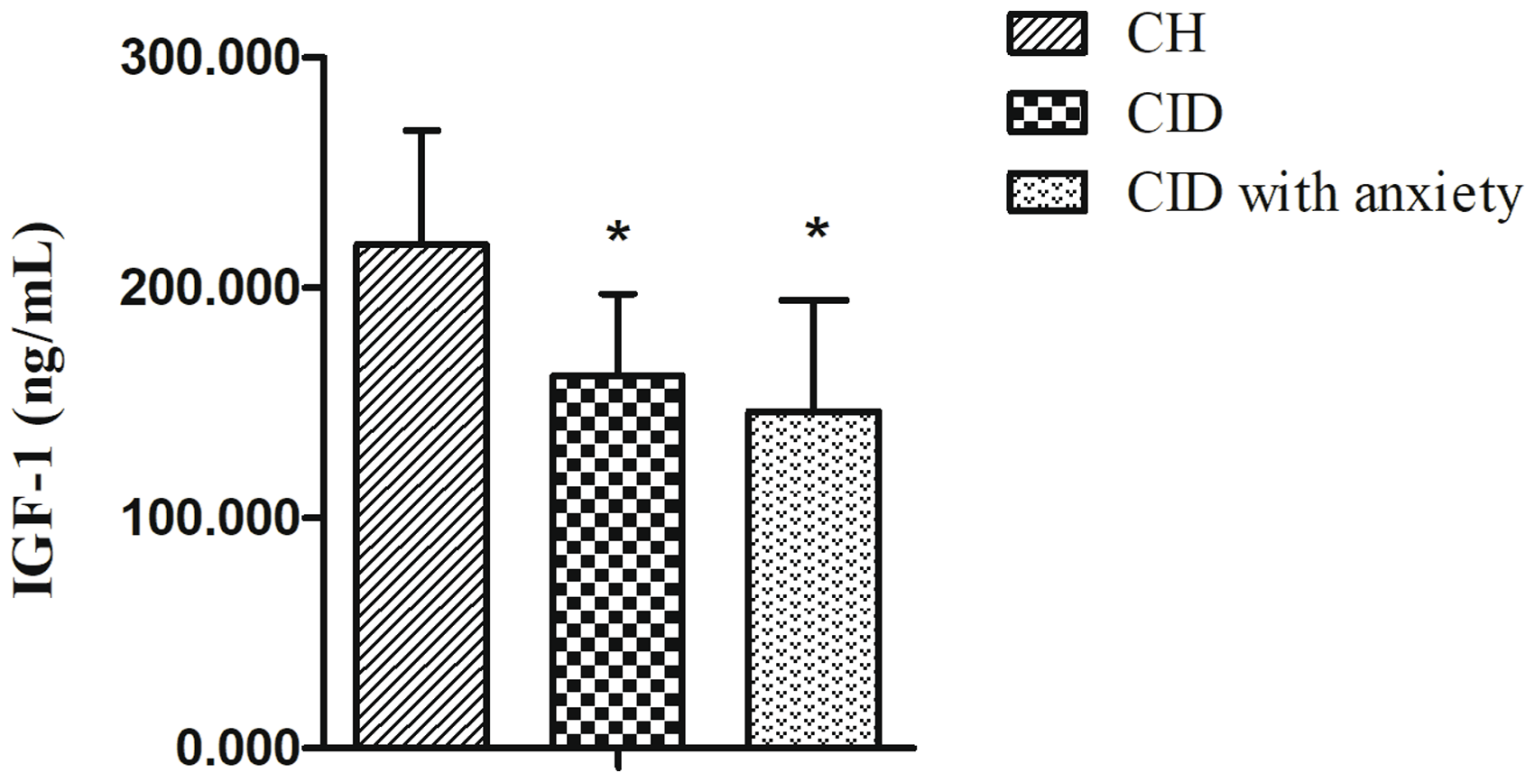
The serum levels of IGF-1 in CID, CID with anxiety patients, and HC. ^*^ indicates statistically different (*p* < 0.05).

A total of 82 patients with CID and 45 age-and sex-matched controls were enrolled in this study. Among them, 65 patients with CID (35 of whom had anxiety) and 36 controls completed PSG and IGF-1 measurements (Figure 1). Baseline characteristics and IGF-1 concentrations are summarized in Table 1 for each group. Male sex, age, marital status, BMI, and PHQ-9 score were very similar in the CID, CID with anxiety, and control groups. The PSQI score and IGF-1 concentration significantly differed among the three groups (both *p* < 0.001). The GAD-7 score in the CID with anxiety group was higher than that in the CID and control groups (both *p* < 0.001). IGF-1 serum concentrations are presented in Figure 2.

**Table 1:** Clinical characteristics and IGF-1 in the patients with CID, CID with anxiety, and HC.

	1. HC (*n* = 36)	2. CID (*n* = 30)	3. CID with anxiety (*n* = 35)	χ^2^/F	*p* value	*Post hoc* Bonferroni (*P*)		
						1 vs.2	1 vs.3	2 vs.3
Male sex	11	12	12	0.648	0.723			
Age (years)				1.346	0.969			
20 ≤ Age < 35	3	4	3					
35 ≤ Age < 45	7	5	7					
45 ≤ Age < 60	20	18	21					
≥60	6	3	4					
Marriage (yes/no)	34/2	27/3	33/2	0.624	0.732			
BMI (kg/m^2^)	24.17 ± 4.03	23.74 ± 3.16	24.91 ± 3.18	0.937	0.395			
Insomnia duration (month)	-	15.3 ± 4.8	14.5 ± 5.4	-	-			
GAD-7 score	3.8 ± 1.7	4.0 ± 1.7	14.8 ± 3.2	242.773	< 0.001^*^	NS	< 0.001^*^	< 0.001^*^
PHQ-9 score	3.8 ± 1.1	4.3 ± 1.6	4.1 ± 1.4	1.604	0.206			
PSQI score	2.5 ± 1.2	13.5 ± 4.6	14.37 ± 3.9	126.777	< 0.001^*^	< 0.001^*^	< 0.001^*^	NS
IGF-1 (ng/mL)	218.537 ± 49.458	161.820 ± 35.461	146.435 ± 48.235	24.773	< 0.001^*^	< 0.001^*^	< 0.001^*^	NS

CID, chronic insomnia disorder; HC, healthy controls; BMI, body mass index; GAD-7, generalized anxiety disorder-7; PHQ-9, patient health questionnaire-9; PSQI, pittsburgh sleep quality index; IGF-1, insulin-like growth factor-1. NS, no significance. ^*^ *p* value < 0.05 (statistically different).

### 3.2. PSG parameters

A comparison of the PSG parameters between the two groups is presented in Table 2. Total sleep time, sleep latency, sleep efficiency, stage N1, stage N3, REM sleep, stage N1 time, stage N2 time, stage N3 time, REM sleep time, and arousal index significantly differed among the groups (all *p* < 0.001).

**Table 2:** The polysomnography parameters in CID, CID with anxiety, and HC.

	1. HC (*n* = 36)	2. CID (*n* = 30)	3. CID with anxiety (*n* = 35)	*F*	*p* value	*Post hoc* Bonferroni (*P*)		
						1 vs.2	1 vs.3	2 vs.3
Total sleep time (min)	393.15 ± 31.93	301.98 ± 66.36	281.88 ± 43.46	53.300	< 0.001^*^	< 0.001^*^	< 0.001^*^	NS
Sleep latency (min)	15.73 ± 3.10	39.87 ± 7.60	41.83 ± 7.41	186.473	< 0.001^*^	< 0.001^*^	< 0.001^*^	NS
Sleep efficiency (%)	90.51 ± 4.02	71.72 ± 11.69	66.93 ± 11.96	57.124	< 0.001^*^	< 0.001^*^	< 0.001^*^	NS
Wake after sleep onset (min)	27.07 ± 20.44	58.24 ± 34.54	84.94 ± 57.75	17.956	< 0.001^*^	0.034	< 0.001^*^	NS
Stage N1 (%)	4.91 ± 1.07	19.55 ± 6.56	21.44 ± 6.40	104.963	< 0.001^*^	< 0.001^*^	< 0.001^*^	NS
Stage N2 (%)	49.83 ± 6.45	51.44 ± 10.03	50.01 ± 7.81	0.358	0.700			
Stage N3 (%)	20.88 ± 4.82	12.27 ± 7.37	10.14 ± 4.70	35.561	< 0.001^*^	< 0.001^*^	< 0.001^*^	NS
REM sleep (%)	24.39 ± 8.33	16.75 ± 8.15	18.934 ± 11.48	5.796	0.004^*^	0.027	NS	NS
Stage N1 time (min)	19.22 ± 4.10	58.88 ± 23.56	60.46 ± 20.83	59.319	< 0.001^*^	< 0.001^*^	< 0.001^*^	NS
Stage N2 time (min)	195.99 ± 31.10	155.63 ± 48.34	142.99 ± 26.78	21.050	< 0.001^*^	< 0.001^*^	< 0.001^*^	NS
Stage N3 time (min)	81.94 ± 19.49	37.49 ± 24.06	28.66 ± 14.30	75.892	< 0.001^*^	< 0.001^*^	< 0.001^*^	NS
REM sleep time (min)	96.00 ± 34.60	50.00 ± 26.72	53.82 ± 35.10	21.107	< 0.001^*^	< 0.001^*^	< 0.001^*^	NS
REM sleep latency	93.75 ± 24.10	117.93 ± 26.59	85.89 ± 32.05	11.449	< 0.001^*^	0.002	NS	< 0.001^*^
REM arousal index (events/h)	1.77 ± 1.36	5.07 ± 3.74	6.28 ± 4.39	16.754	< 0.001^*^	< 0.001^*^	< 0.001^*^	NS
Total arousal index (events/h)	5.95 ± 3.30	16.01 ± 4.86	19.50 ± 5.72	60.381	< 0.001^*^	< 0.001^*^	< 0.001^*^	0.039
AHI (per hour)	2.13 ± 1.60	2.31 ± 0.99	2.23 ± 1.04	0.162	0.850			
PLMI (events/h)	0.99 ± 0.84	0.82 ± 1.01	0.91 ± 0.90	0.268	0.765			
Mean SaO_2_ (%)	95.83 ± 1.54	94.97 ± 1.81	95.23 ± 1.78	2.279	0.108			
Minimum SaO_2_ (%)	87.86 ± 5.28	86.33 ± 6.09	86.57 ± 5.44	0.743	0.478			
End-tidal CO_2_ (mmHg)	35.34 ± 2.55	35.45 ± 2.20	35.77 ± 2.53	0.298	0.743			

CID, chronic insomnia disorder; HC, healthy controls; REM, rapid eye movement; Stage N1, stage 1 non-REM; Stage N2, stage 2 non-REM; Stage N3, stage 3 non-REM; AHI, apnea-hypopnea index; PLMI, periodic limb movement index; SaO_2_, arterial oxygen saturation. NS, no significance. ^*^p value < 0.05 (statistically different).

### 3.3. Correlation analysis for IGF-1 concentrations with GAD-7, PSQI, and PSG parameters in CID and CID with anxiety groups

**Figure 3:**
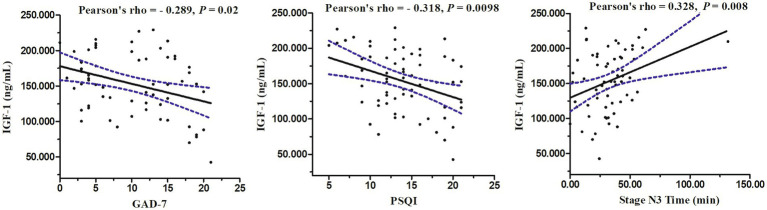
Relations between serum levels of IGF-1 and GAD-7 score, PSQI score, and stage N3 time in PSG.

Using Pearson correlation analysis, the IGF-1 concentration in patients with CID was significantly correlated with the GAD-7 score (*r* = −0.289, *p* = 0.02), PSQI score (*r* = −0.318, *p* = 0.0098), and stage N3 time (*r* = 0.328, *p* = 0.008; Figure 3).

### 3.4. Univariable and multivariable analysis actors affecting IGF-1 serum levels in CID and CID with anxiety groups

Table 3 summarizes the univariate and multivariable regression analysis of factors affecting the IGF-1 serum concentration. In the univariate model, IGF-1 concentration was associated with age (*p* = 0.017), GAD-7 score (*p* = 0.005), PSQI score (*p* = 0.003), and stage N3 time (*p* = 0.007). However, none of these or any other variables associated with IGF-1 concentration upon multivariable analysis.

**Table 3:** Univariable and multivariable analysis for the IGF-1.

Factors	IGF-1, ng/mL			
	Univariable analysis		Multivariable analysis	
	*β*	*P*	*β*	*P*
Male sex	−0.045	0.721		
Age (years)	−0.295	0.017 ^ab^	−0.167	0.154
BMI (kg/m^2^)	0.204	0.104		
Insomnia duration	−0.164	0.192		
GAD-7 score	−0.346	0.005^ab^	−0.229	0.053
PHQ-9 score	0.185	0.140		
PSQI score	−0.366	0.003 ^ab^	−0.227	0.062
Total sleep time (min)	0.169	0.179		
Sleep latency (min)	0.022	0.863		
Sleep efficiency (%)	0.183	0.145		
Wake after sleep onset (min)	−0.132	0.294		
Stage N1 time (min)	0.245	0.256		
Stage N2 time (min)	0.007	0.956		
Stage N3 time (min)	0.333	0.007^ab^	0.165	0.176
REM sleep time (min)	−0.042	0.742		
REM sleep latency	0.110	0.381		
REM arousal index (events/h)	0.079	0.534		
Arousal index (events/h)	−0.055	0.665		
AHI (per hour)	−0.002	0.986		
PLMI (events/h)	0.058	0.648		
Mean SaO_2_ (%)	−0.086	0.494		
Minimum SaO_2_ (%)	−0.070	0.579		
End-tidal CO_2_ (mmHg)	0.158	0.210		

IGF-1, insulin-like growth factor-1; BMI, body mass index; GAD-7, generalized anxiety disorder-7; PHQ-9, patient health questionnaire-9; PSQI, pittsburgh sleep quality index; REM, rapid eye movement; Stage N1, stage 1 non-REM; Stage N2, stage 2 non-REM; Stage N3, stage 3 non-REM; AHI, apnea-hypopnea index; PLMI, periodic limb movement index; SaO_2_, arterial oxygen saturation. ^a^Nominally significant values (*p* < 0.1) included in the multivariable model; ^b^*p* value < 0.05 (statistically different).

## 4. Discussion

In this study, the IGF-1 serum concentration in patients with CID was lower than that in patients without CID. Moreover, the IGF-1 concentration was negatively correlated with participants’ anxiety score and sleep quality and positively correlated with stage N3 time during PSG, which indicates that IGF-1 may play an important role in insomnia and mood disorders.

Chronic sleep deprivation reportedly reduces the total IGF-1 concentration in rats (20, 21). Chennaoui et al. also discovered that the IGF-1 system responds to sleep deprivation, with serum IGF-1 concentrations decreasing after 25 h of sleep deprivation and increasing again after a night of recovery (7). Kimura et al. reported that decreases in the symptoms of patients with circadian rhythm sleep–wake disorders were associated with increased serum concentrations of IGF-1 (22). Although the results differ among those studies, they all suggest that sleep deprivation is related to IGF-1. Disruptions during sleep, such as insomnia, can affect GH and IGF-1 concentrations, because GH is preferentially released during slow-wave sleep. In addition, a delay in sleep can result in a decrease in GH secretion (23). Our results appear to support the link between IGF-1 and insomnia and are in line with those of other studies on IGF-1. Besides, because of the small size of the age subgroups in our study, further work is needed to confirm these data.

Several potential mechanisms underlying decreased IGF-1 concentrations in patients with CID may be involved. First, as mentioned above, although GH secretion occurs in pulses throughout the day, slow-wave sleep after sleep onset is associated with particularly large bursts of GH secretion. Secretion of growth-promoting hormone cells also increases during slow-wave sleep. The relationship between nocturnal GH release and slow wave activity suggests that it can reflect the GH release hormone activity. GH-releasing hormone may promote sleep and reduce the awakening threshold of REM sleep. Symptoms of insomnia include difficulty falling asleep, decreased total sleep time, reduced slow-wave sleep time, and increased sleep fragmentation (5). These symptoms cause a decrease in GH secretion, which, in turn, causes a decrease in the IGF-1 concentration. This was supported by the correlation between IGF-1 concentration and the N3 stage in this study. Additionally, the hypothalamic–pituitary–adrenal (HPA) axis is activated in patients with CID, which has a suppressant effect on the GH-IGF-1 axis (24). In particular, cortisol secretion is negatively correlated with GH secretion, which subsequently affects the IGF-1 concentration (25). Second, patients with CID have HPA-axis dysfunction, exhibiting an increase in the release of adrenocorticotropic hormone, increased sympathetic nervous system activity, and increased inflammatory cytokine concentration (24, 26). Experimental studies have also suggested that altered sleep may impact concentrations of inflammatory markers, such as interleukin-6 (IL-6), tumor necrosis factor-α (TNF-α), and C-reactive protein (27). Proinflammatory cytokines may suppress the GH/IGF-1 axis and decrease both circulatory and tissue concentrations of IGF-1 (28–30).

Furthermore, IGF-1 is a neuroprotective agent, involved in brain development and survival (31). Thus, IGF-1 may play an important role in the amelioration of anxiety and memory deficits (32). A higher intraindividual IGF-1 concentration is reportedly associated with a better mood. Previous studies have revealed that IGF-1 concentrations are negatively correlated with anxiety levels (33, 34), which is consistent with our results. However, in other studies, individuals with depressive and/or anxiety disorders had higher IGF-1 concentrations than those without such disorders, which may indicate a response mechanism to counteract the impaired neurogenesis (35). Although the IGF-1 concentration was appeared lower in the CID with anxiety group than in the CID group in our study, the difference was not statistically significant. We plan to conduct further studies with larger samples to verify this result. Based on our results, we hypothesize that the HPA axis plays a role in the relationship between the GAD-7 score and the IGF-1 concentration. A dysfunctional HPA axis has been implicated in the pathogenesis of anxiety disorders. Proinflammatory cytokines, including IL-6 and TNF-α, have been implicated in the etiologies of clinical anxiety disorders, which may affect the IGF-1 concentration (36). However, anxiety may affect IGF-1 in diverse ways, and further clarity will require further investigation.

This study has several limitations. First, it was an observational study without in-depth investigation into underlying mechanisms. We are currently monitoring IGF-1 concentrations in patients with CID after treatment, in order to elucidate these mechanisms. Second, previous studies have revealed that orexin neurons are modulated by IGF-1 (37), which may play an important role in the pathophysiology of insomnia. This aspect was not considered in the current study. Therefore, we will focus on the correlation between hypothalamic secretion and IGF-1 in the future. In addition, our study was limited by the small sample, and future studies will be conducted with larger samples to allow age stratification, which should provide a stronger evidence base for the clinical diagnosis and treatment of insomnia and mood disorders.

## 5. Conclusion

In conclusion, the serum IGF-1 concentration in patients with CID was lower than that in participants without CID, negatively correlated with anxiety score and sleep quality, and positively correlated with stage N3 time during PSG. This indicates a potentially important role of IGF-1 in insomnia and emotional disorders. Further studies are needed to examine the relationship between IGF-1 and insomnia and their influence on accompanying symptoms.

## Data availability statement

The raw data supporting the conclusions of this article will be made available by the authors, without undue reservation.

## Ethics statement

The studies involving human participants were reviewed and approved by the Ethics Committee of the First Hospital of Jilin University. The patients/participants provided their written informed consent to participate in this study.

## Author contributions

YZ wrote the manuscript. QS and HL conducted the data acquisition and data analysis. YW and DW prepared the figures. ZW managed the study and edited the final manuscript. All authors contributed to the article and approved the submitted version.

## Funding

The work was supported by the National Natural Science Foundation of China (Grant number 82071489), the National key research and development program of China (Grant number 2022YFC2503904), and the Foundation of the Department of Science and Technology of Jilin Province (Grant Number 20200404093YY) to ZW.

## Conflict of interest

The authors declare that the research was conducted in the absence of any commercial or financial relationships that could be construed as a potential conflict of interest.

## Publisher’s note

All claims expressed in this article are solely those of the authors and do not necessarily represent those of their affiliated organizations, or those of the publisher, the editors and the reviewers. Any product that may be evaluated in this article, or claim that may be made by its manufacturer, is not guaranteed or endorsed by the publisher.
